# Residues R177 and S178 of the human Na^+^/H^+^ antiporter NHA2 are involved in its inhibition by the flavonoid phloretin

**DOI:** 10.1002/1873-3468.15089

**Published:** 2024-12-31

**Authors:** Olga Zimmermannova, Martin Kubeš, Tereza Przeczková, Gal Masrati

**Affiliations:** ^1^ Laboratory of Membrane Transport Institute of Physiology of the Czech Academy of Sciences Prague 4 Czech Republic; ^2^ Department of Biochemistry and Molecular Biology, George S. Wise Faculty of Life Sciences Tel‐Aviv University Israel

**Keywords:** human NHA2, Na^+^/H^+^ antiporter, phloretin inhibition, yeast

## Abstract

The *Homo sapiens* Na^+^/H^+^ antiporter NHA2 (*SLC9B2*) transports Na^+^ or Li^+^ in exchange for protons across cell membranes, and its dysfunction results in various pathologies. The activity of *Hs*NHA2 is specifically inhibited by the flavonoid phloretin. Using bioinformatic modeling, we predicted two amino acids (R177 and S178) as being important for the binding of phloretin to the *Hs*NHA2 molecule. Functional expression of *Hs*NHA2 in *Saccharomyces cerevisiae* and its site‐directed mutagenesis revealed that while the R177T mutation resulted in an antiporter that was less sensitive to phloretin, the S178T mutation enhanced the inhibitory effect of phloretin on *Hs*NHA2. Our data corroborate the transport properties of *Hs*NHA2 and its interactions with an inhibitor and can be helpful for the development of new therapeutics targeting this antiporter and its pleiotropic physiological functions.

## Graphical abstract

The human Na^+^/H^+^ antiporter NHA2 transports Na^+^ or Li^+^ in exchange for protons across cell membranes, and its dysfunction results in various pathologies, e.g., fertility disorders, hypertension, or diabetes. This work provides the molecular characterization of interactions of NHA2 with its specific inhibitor phloretin, which can be helpful for the development of new therapeutics targeting this antiporter and its pleiotropic physiological functions.

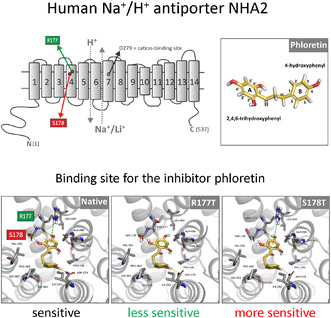

## Abbreviations

**CPA**, cation/proton antiporters

**DMSO**, dimethylsulfoxide

**GFP**, green fluorescent protein

***NHA1***, gene encoding the yeast Na^+^, K^+^/H^+^ antiporter

**NHA2**, human Na^+^/H^+^ antiporter (SLC9B2)

**NHE**, Na^+^/H^+^ antiporters (SLC9A family)

**OD**, optical density

**SDS**, sodium dodecyl‐sulfate

**TEA**, triethanolamine

**TMS**, transmembrane segment

***TPS1***, gene encoding the yeast trehalose‐6‐P synthase

**YNB**, yeast nitrogen base

**YPD**, yeast extract peptone dextrose

Cation/H^+^ antiporters (CPAs, SLC9 family) mediate the exchange of monovalent cations, mainly Na^+^ and K^+^, for one or two protons across the cell membrane. They are essential for the maintenance of intracellular pH and the concentrations of monovalent cations in organisms of all kingdoms, from bacteria to mammals [1]. In humans, there are 13 isoforms divided into three subfamilies—nine NHE proteins (SLC9A; NHE1‐9), two NHA proteins (SLC9B; NHA1‐2), and two of the SLC9C subfamily (NHE10‐11) [2, 3]. There is a growing list of pathologies related to the malfunctioning of particular members of Na^+^/H^+^ antiporters, e.g., hypertension, autism spectrum disorder, metabolic diseases (diabetes), or cancer, which demonstrates the importance of Na^+^/H^+^ antiporters for human health. Therefore, understanding the molecular mechanisms governing ion transport and the binding of inhibitors is important in the development of therapeutics targeting particular isoforms [3].

A member of the SLC9B subfamily encoded by the NHA2 gene is a Na^+^(Li^+^)/H^+^ antiporter, which was found to be involved in the regulation of intracellular pH, sodium homeostasis, and cellular volume [3]. Impaired activity of NHA2 leads to various pathologies ranging from metabolic to fertility disorders [4, 5]. Linkage studies associated the locus with the NHA2 gene (a human chromosomal region 4q24) with hypertension [6], or with type 2 diabetes [7]. *In vitro* as well as *in vivo* studies found NHA2 to be a critical player for insulin secretion in pancreatic β‐cells [8]. In mice, the loss of NHA2 function exacerbated obesity‐ and aging‐induced glucose intolerance [9]. In the kidney, it is critical for sodium reabsorption in the distal convoluted tubules and blood pressure homeostasis [10, 11, 12, 13]. In addition, NHA2 depletion significantly inhibited osteoclast differentiation *in vitro* [14, 15, 16]. However, its physiological role *in vivo* in this type of cell is still unclear [17, 18].

**Fig. 1:**
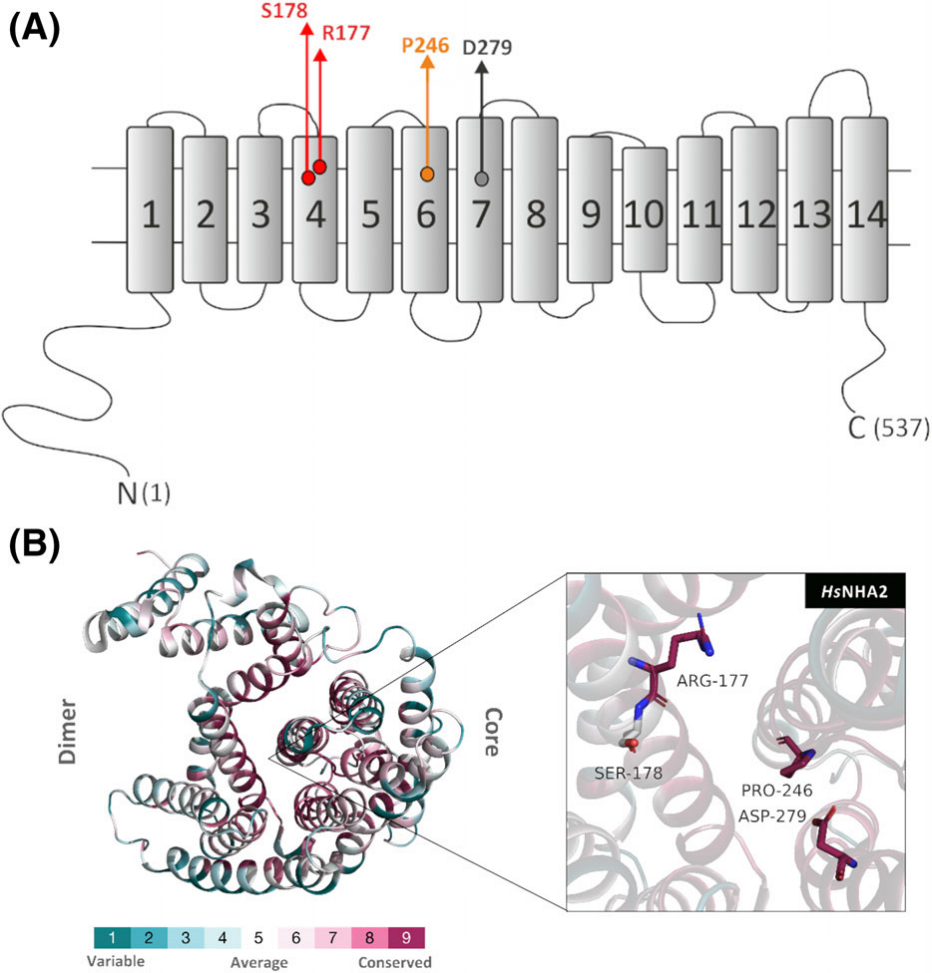
Position of R177 and S178 within the structure of *Hs*NHA2. Topological model (A) and 3D structure (B) of *Hs*NHA2 with 14 TMS. The positions of the 4 amino acids that are important for this study are highlighted (D279—cation binding site, P246—a residue important for its cation selectivity and phloretin binding [22], R177 and S178—putative binding sites for phloretin studied in this work). In (B), residues 1 to 537 are colored based on the ConSurf server evolutionary conservation scale, with cyan to maroon representing variable to conserved amino acids, respectively.

**Fig. 2:**
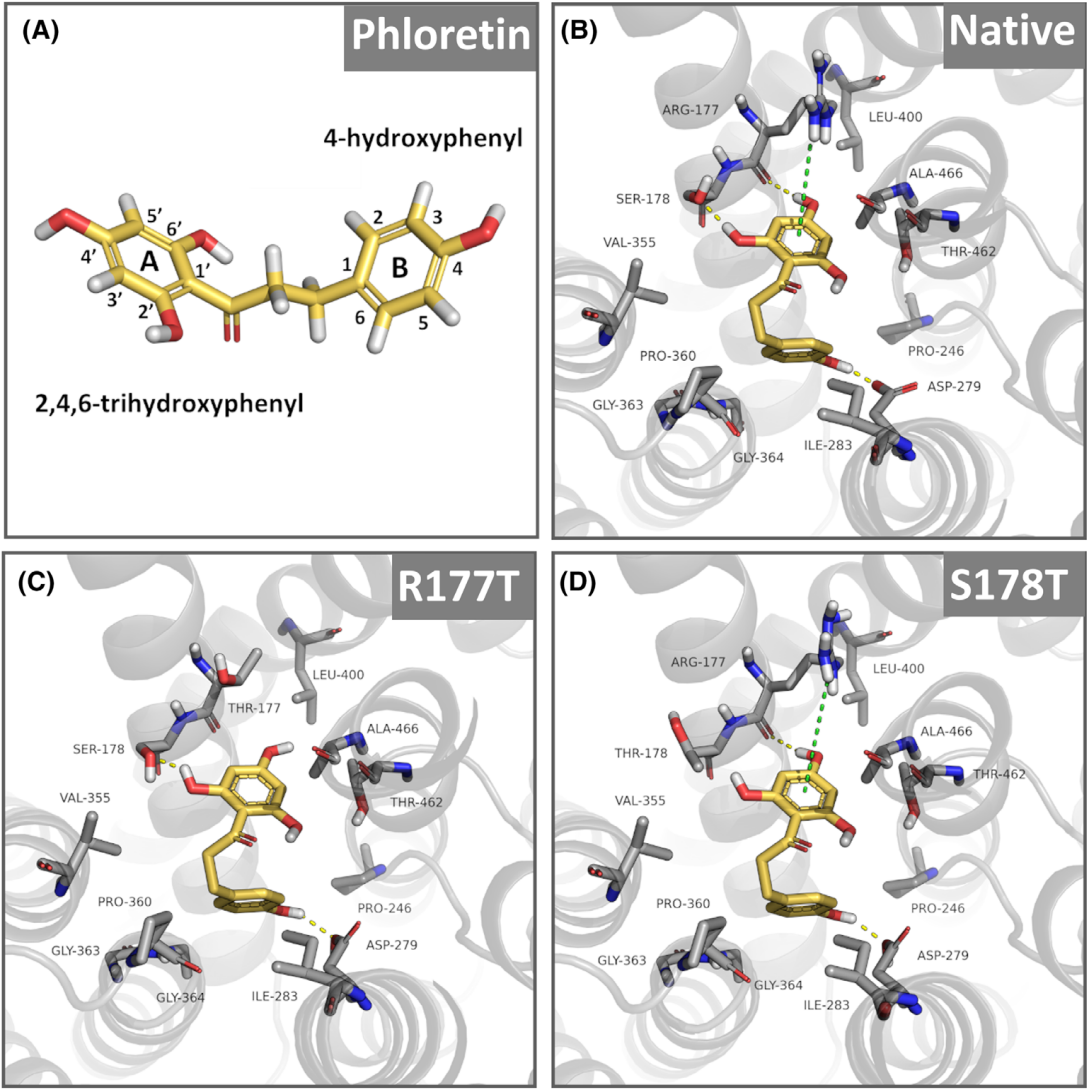
Model of phloretin binding to *Hs*NHA2. Phloretin, shown in yellow, was docked to the crystal structure of human NHA2 (PDB entry 7B4L; native or R177T or S178T mutated versions) as described in Materials and methods and in [22]. Residues within a radius of 5 Å from phloretin are shown as sticks. (A) Structure of phloretin with the commonly named rings A (2,4,6‐trihydroxyphenyl) and B (4‐hydroxyphenyl). (B) In the native protein, phloretin is electrostatically stabilized by three amino acids. The 2,4,6‐trihydroxyphenyl ring interacts *via* hydrogen bonds with the side chain hydroxyl of S178 and the backbone carbonyl of R177. It also forms a cation‐π interaction with the side chain guanidinium of R177. The 4‐hydroxyphenyl ring hydrogen bonds with D279. (C) The R177T mutation could disrupt the highly favorable cation‐π interaction between the phloretin's 2,4,6‐trihydroxyphenyl ring and arginine's side chain guanidium group. (D) In the S178T mutant, the threonine side chain can theoretically adopt a different rotamer that maintains the native hydrogen bond with phloretin, originally contributed by S178.

NHA2 mediates an electroneutral efflux of sodium or lithium cations in exchange for external protons across the membrane [4, 19]. Human NHA2 (*Hs*NHA2) consists of 537 amino acids with a 14 transmembrane segments (TMS) topology with both its N and C terminus in the cytosol and aspartate 279 in the TMS7 as a cation‐binding site (Fig. 1A; Protein Data Bank 7B4M/L). A 3D structure has also been recently resolved for the bison homolog of NHA2 [20]. In contrast to plasma membrane NHE antiporters of the SLC9A subfamily, the activity of *Hs*NHA2 is insensitive to amiloride and sensitive to phloretin [6, 21, 22]. Phloretin (Fig. 2A), a flavonoid abundant in apples, is a highly flexible molecule with the ability to bind to biological macromolecules and with high pharmacological and pharmaceutical potential due to its antimicrobial, antioxidant, anti‐inflammatory, and anticancer activities [23].

Recently, we optimized the functional expression of *Hs*NHA2 in *S. cerevisiae* cells and obtained a highly valuable experimental model useful for the determination of effects of *Hs*NHA2 mutations that alter its substrate specificity, transport activity, or stability [22]. We clearly demonstrated that the ability of the native *Hs*NHA2 to improve LiCl as well as NaCl tolerance of yeast cells at pH 4.0 highly decreased in the presence of phloretin [22]. We also revealed that the inhibitory effect of phloretin decreased with mutations of proline 246 (TMS6; Fig. 1A) in the core of the protein (Fig. 1B) [22]. This experimental approach supported by molecular modeling suggested that a molecule of the inhibitor phloretin binds to the catalytic center of *Hs*NHA2 close to the cation‐binding site and proline 246 ([22]; Fig. 2B).

Detailed knowledge of the mechanisms of interaction of Na^+^/H^+^ antiporters with inhibitors is crucial for the development of new drugs and a general understanding of monovalent ion transport across the membrane. The main aim of this work was to elucidate the importance of two amino acid residues (R177 and S178; TMS4, Fig. 1) predicted by molecular modeling to be involved in the interaction with phloretin (Fig. 2B). Consistent with the modeling, our experimental results show that the binding site for phloretin is most likely located in the central part of *Hs*NHA2. While mutations of R177A/T appear to reduce the inhibitory effect of phloretin on *Hs*NHA2, introduction of the β‐branched polar amino acid threonine instead of S178 enhanced its effect on *Hs*NHA2 transport activity.

## Materials and methods

### Yeast strains and growth media

An alkali‐metal‐cation‐sensitive *S. cerevisiae* W303‐1A derivative – BW31 (*ena1Δ*::*HIS3*::*ena4Δ nha1Δ*::*LEU2*) [24], which lacks genes encoding plasma membrane Na^+^ and Li^+^ exporters (Ena ATPases and Nha1 antiporter), was used to characterize *Hs*NHA2 activity. Yeast cultures were routinely grown in YPD (Formedium, Norfolk, UK) or YNB media (Difco, Sparks, MD, USA) at 30 °C. YNB‐based media (referred in this work as YNB‐Pro) contained 0.17% YNB without amino acids and ammonium sulphate, 0.1% proline, and 2% or 4% glucose. Proline was used instead of ammonium sulphate as a nitrogen source. A mixture of appropriate auxotrophic supplements (adenine, tryptophan, leucine, and histidine—each at a final concentration of 20 μg·mL^−1^) was added after autoclaving. YPD media were supplemented with extra adenine (final concentration 20 μg·mL^−1^) to avoid additional spontaneous mutagenesis caused by the *ade2* mutation [25]. Solid media were prepared by adding 2% or 3% (w/v) agar.

### Plasmids and site‐directed mutagenesis

For the expression of *Hs*NHA2 antiporter non‐tagged or tagged with GFP at its N terminus in yeast cells, two previously described [22] plasmids were used, p*Hs*NHA2t and pGFP‐*Hs*NHA2t, respectively, i.e., multi‐copy plasmids in which *Hs*NHA2 or GFP‐*Hs*NHA2 is expressed under the control of the *S. cerevisiae NHA1* promoter and *TPS1* terminator. Five mutated versions with point mutations (R177A/T, S178A/T, R177A + S178A) derived from both plasmids were prepared by using a QuikChange XL Site‐Directed Mutagenesis kit (Agilent Technologies, Santa Clara, CA, USA) and two overlapping complementary oligonucleotides containing the corresponding nucleotide changes. The accuracy of introduced mutations was confirmed by sequencing. Previously prepared plasmids p*Hs*NHA2t(P246T) and pGFP‐*Hs*NHA2t(P246T) [22] for the expression of *Hs*NHA2 with the point mutation P246T were used as controls.

### Growth assays

To estimate the cell tolerance to various alkali‐metal cations, YNB‐Pro media were supplemented with LiCl or NaCl at the indicated concentration. HCl or TEA (triethylamine) were used to adjust the pH to 4.0 or 7.0, respectively. To test the inhibitory effect of phloretin on *Hs*NHA2 activity and its variants, media were supplemented with a solution of phloretin (Merck, Darmstadt, Germany, cat. no. P7912) at the indicated concentrations (100 mm stock solution of phloretin was prepared in DMSO). Tenfold serial dilutions of cell suspensions in water (OD_600_ = 2, Eppendorf BioPhotometer, Hamburg, Germany) were spotted on plates with indicated concentrations of salts and/or phloretin, and growth was monitored for 2–7 days. At least four independent transformants were tested for each mutated version of *Hs*NHA2. Representative results of at least three independent experiments are shown.

### Fluorescence microscopy

Microscopic images of yeast cells were acquired with an Olympus BX53 microscope with an Olympus DP73 camera (Olympus, Tokio, Japan). A Cool LED light source with 460 nm excitation and 515 nm emission was used to visualize GFP‐*Hs*NHA2 and its mutated variants. Cells containing the corresponding multi‐copy plasmids were grown overnight in YNB‐Pro with 4% glucose and supplemented with adenine and tryptophan (final concentration 40 μg·mL^−1^), and leucine and histidine (final concentration 20 μg·mL^−1^). Cells were observed when they reached the exponential phase of growth (OD_600_≈0.4–0.6). A Nomarski prism was used for whole‐cell images. The experiment was repeated three times, and representative pictures are shown.

### Protein extraction and immunoblotting

Cells expressing the native or mutated GFP‐*Hs*NHA2 were grown in YNB plus 4% glucose to OD_600_ = 0.6, and the total extract of proteins was prepared as previously described [22]. Protein quantification was performed using RC DC™ Protein Assay (Bio‐Rad, Hercules, CA, USA). For SDS/PAGE (10% polyacrylamide), an amount corresponding to 120 mg of protein extracts was loaded for each sample and transferred to nitrocellulose membranes (Trans‐Blot Turbo 0.2 mm Nitrocellulose) using a Trans‐Blot Turbo Transfer System (Bio‐Rad). Membranes were incubated overnight at 4 °C with a 1 : 500 dilution of mouse monoclonal antibody against GFP (Roche, Basel, Switzerland). After that, a 1 : 10 000 dilution of secondary antimouse IgG‐horseradish peroxidase (GE Healthcare, Chicago, IL, USA) was used. Immunoreactive proteins were visualized with a Clarity Max Western ECL substrate kit (Bio‐Rad) in ChemiDoc imaging systems (Bio‐Rad). To detect Pgk1 protein, the same membranes used for GFP detection were probed again by being incubated for 1 h at room temperature with a 1 : 20 000 dilution of mouse monoclonal antibody against Pgk1 (Abcam, Cambridge, UK) and the signal was analyzed in the same way as for GFP. Representative results of three independent experiments are shown.

### Modeling of phloretin binding to *Hs*NHA2 and its mutated versions

A similar procedure as we used previously [22], using Glide [26], was applied for docking of a molecule of phloretin to the crystal structure of human NHA2 (PDB entry 7B4L) and its mutated versions R177T or S178T. Briefly, mutations to the protein were introduced using pymol (The PyMOL Molecular Graphics System, version 3.0 Schrödinger, LLC., New York, NY, USA). The crystal structure and mutated versions were initially prepared for docking using the Protein Preparation Wizard [27]. The protonation states of all residues were determined with PROPKA3 [28], with the exception of D278 and D279, which were modeled in all combinations of protonation states. Figure 2B–D arbitrarily shows both D278 and D279 in their deprotonated state. The docking poses were scored and selected according to the extra precision scoring function (XP) [29]. The docking score for the *Hs*NHA2‐phloretin complex varies between −4.9 to −6.4 kcal·mol^−1^ depending on the protonation states of D278 and D279. It is important to highlight that, despite being expressed in energy units, docking scores do not directly correspond to actual Δ*G* values. This is because they are derived from a single conformation of the ligand‐protein complex and thus neglect dynamic and entropic contributions related to small molecule binding. The figures were produced using pymol.

## Results

### R177 and S178 are putative phloretin binding sites in *Hs*NHA2

Mutagenesis studies of P246 in *Hs*NHA2, located in TMS6 (Fig. 1), i.e., one of the two unwound transmembrane segments that create the X‐shaped structure in CPAs' core domain [20], found that its replacement with the polar‐branched threonine resulted in an antiporter that transports Na^+^ better than Li^+^ [22]. Moreover, and in contrast to the native antiporter, *Hs*NHA2 harboring the point mutations P246A/S/T also exhibited activity at a higher external pH (a pH range of 4.0–5.5 vs. 4.0–7.4) [22]. Alterations at the position of P246 also significantly decreased the inhibitory effect of phloretin [22]. All these data obtained by the heterologous expression of *Hs*NHA2 in *S. cerevisiae* identified proline 246 as a crucial residue involved in the ability to recognize and transport particular substrates (ion selectivity), and most likely also influencing the capacity to transport protons. The results also indicated that phloretin acts directly in the core domain of *Hs*NHA2, and mutations of P246 possibly cause inhibitor resistance by altering the binding site's geometry near the substrate/inhibitor‐binding pocket. Our model depicting the binding of phloretin to the *Hs*NHA2 (Fig. 2) predicted that in the native molecule of *Hs*NHA2, phloretin binds and directly interacts with the highly conserved D279 in *Hs*NHA2's cation‐binding site and is stabilized by interactions with two other amino acids, R177 and S178 (Fig. 2B; [22]). The 4‐hydroxyphenyl ring (Fig. 2A, ring B) hydrogen bonds with the cation‐binding D279, and the 2,4,6‐trihydroxyphenyl ring of the phloretin (Fig. 2A, ring A) interacts *via* hydrogen bonds with the side chain hydroxyl of S178 and the backbone carbonyl of R177 (Fig. 2B). Modeling also shows a possible cation‐π interaction of the 2,4,6‐trihydroxyphenyl ring with the side chain guanidinium of R177 (Fig. 2B). In this work, we experimentally studied the involvement of R177 and S178 in the interaction of *Hs*NHA2 with the molecule of phloretin. To characterize the effect of these two amino acid residues on the activity of *Hs*NHA2 in the absence or presence of phloretin, we used the same methodology and optimized functional expression system of *Hs*NHA2 in yeast cells as we used previously [22]. In all further experiments, *Hs*NHA2 was expressed from the multi‐copy plasmid, p*Hs*NHA2*t* or pGFP‐*Hs*NHA2*t* (for the expression of *Hs*NHA2 with the GFP tag at the N terminus), under the control of a weak and constitutive *S. cerevisiae NHA1* promoter and *TPS1* terminator in a salt‐sensitive strain BW31, which lacks the two main plasma membrane Na^+^ and Li^+^ exporters (*nha1Δ ena1‐4Δ*) [22]. To experimentally prove the importance and function of the two predicted interacting residues, R177 and S178, we prepared five mutated versions of *Hs*NHA2 by site‐directed mutagenesis. At the indicated positions, we either introduced the hydrophobic alanine to disrupt polar bonds (R177A, S177A), or branched polar threonine (R177T, S178T), because it had the highest effect on phloretin sensitivity for P246 [22]. We also tested the double‐mutated version with both residues replaced with alanine (R177A + S178A). Mutated antiporters were expressed in the BW31 strain from the corresponding p*Hs*NHA2t or pGFP‐*H*sNHA2t plasmids, and the transport activity as well as localization and protein stability of mutated *Hs*NHA2 were determined.

### Mutations of R177 and S178 impact the transport properties, localization and protein stability of *Hs*NHA2

**Fig. 3:**
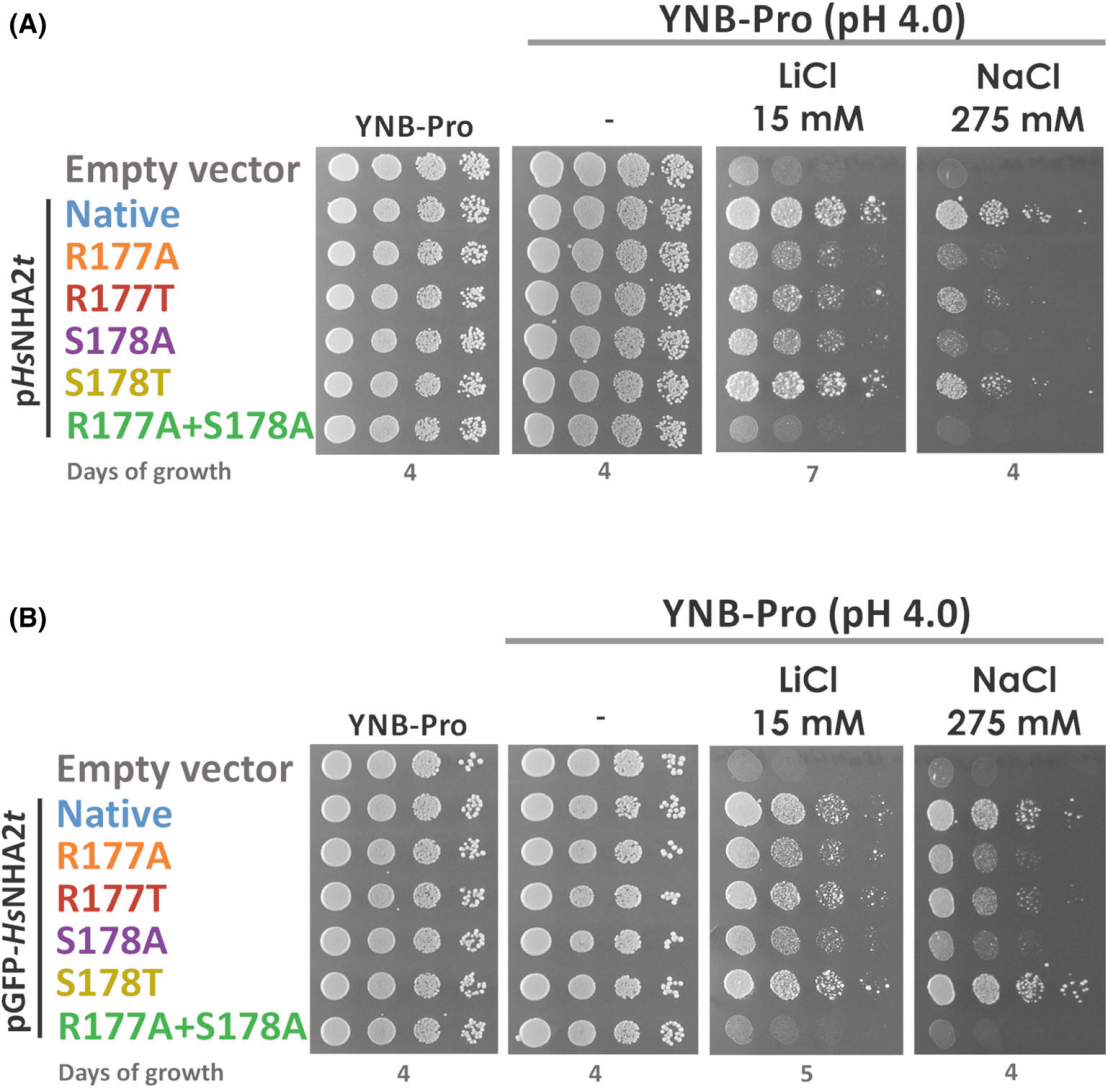
Characterization of *Hs*NHA2 with point mutations at residues R177 or S178. The salt tolerance of *S. cerevisiae* BW31 cells containing the empty vector or expressing the native *Hs*NHA2 or one of five *Hs*NHA2 mutated versions (R177A, R177T, S178A, S178T or R177A + S178A) from p*Hs*NHA2t (A) or pGFP‐*Hs*NHA2t (B) plasmids. Cells were grown on YNB‐Pro or YNB‐Pro plates with pH adjusted to 4.0 and supplemented with LiCl or NaCl as indicated. Plates were incubated at 30 °C and photographed on the indicated day. The experiment was repeated three times, and representative results are shown.

First, we estimated the effect of R177A/T and S178A/T mutations on the substrate specificity and transport activity of *Hs*NHA2. Cells expressing mutated versions of *Hs*NHA2 were tested for their tolerance to LiCl or NaCl at pH 4.0 or 7.0 in comparison with cells transformed with the empty vector or expressing the native *Hs*NHA2 antiporter (Fig. 3). The expression of mutated *Hs*NHA2 was not toxic for cells, as all transformants grew as well as cells containing the empty vector or the native *Hs*NHA2 on control plates without salts (Fig. 3). Neither the native nor mutated *Hs*NHA2 improved the tolerance of cells to salts at pH 7.0 (not shown). At pH 4.0, the growth of cells with the empty vector was severely inhibited on plates supplemented with LiCl or NaCl, but the presence of the native *Hs*NHA2 improved the growth of cells in the presence of both LiCl and NaCl according to its substrate specificity and in accordance with our previous results (Fig. 3; [22]). Compared to the native antiporter, mutated versions R177A/T and S178A were less efficient exporters, especially for Na^+^, as reflected by worse cell growth with these versions in the presence of both NaCl and LiCl in comparison with cells expressing the native *Hs*NHA2 (Fig. 3A). Expression of *Hs*NHA2 with the S178T mutation, i.e., a substitution of a non‐branched polar amino acid for a β‐branched polar amino acid, had only slight negative effect on the cell salt tolerance (Fig. 3A). The R177A + S178A double mutant was completely non‐functional, as its presence did not increase cell tolerance to either LiCl or NaCl compared to cells with the empty vector (Fig. 3A). For both amino acids, R177 and S178, replacing either of them with a threonine residue had a less negative effect than replacing them with alanine (Fig. 3A). Similar phenotypes were observed for cells expressing the same mutated versions of *Hs*NHA2 tagged at the N terminus with GFP (Fig. 3B). In this case, the expression of a GFP‐tagged S178T variant of *Hs*NHA2 improved the tolerance of cells to NaCl and LiCl slightly better than the native *Hs*NHA2 (Fig. 3B).

**Fig. 4:**
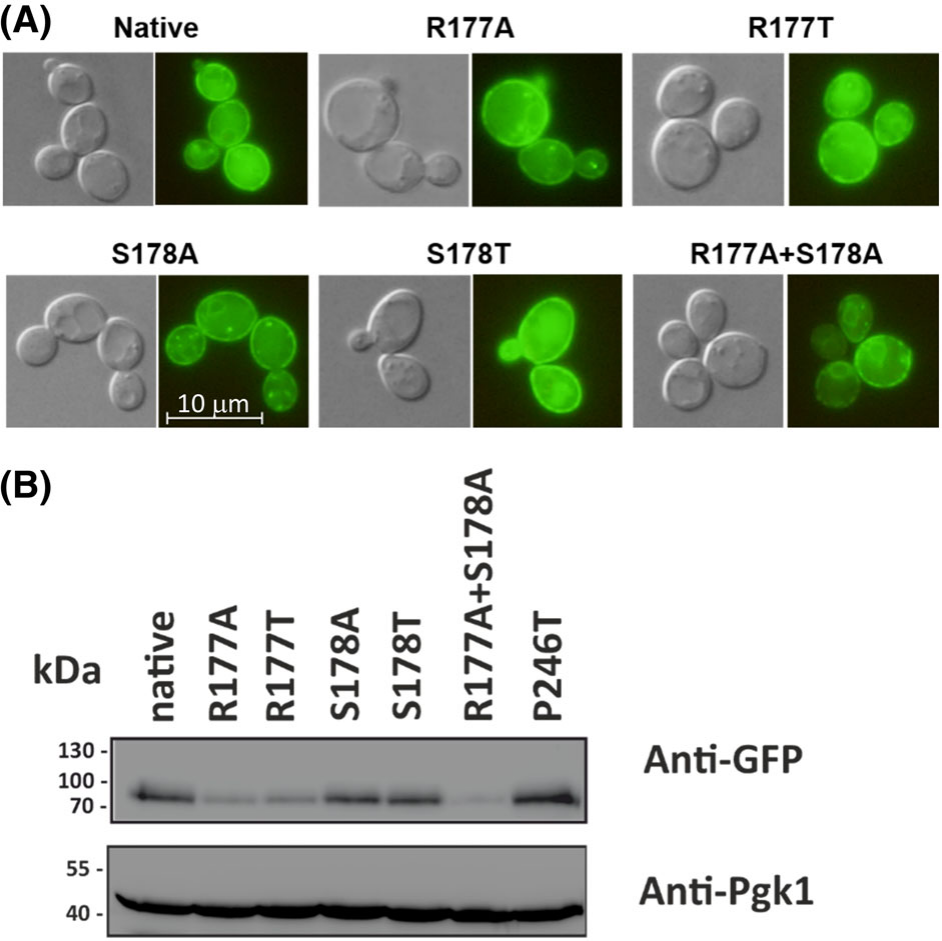
Detection of expression of *Hs*NHA2 with point mutations at residues R177 or S178 in yeast cells. Localization (A) and immunodetection (B) of N‐terminally GFP‐tagged *Hs*NHA2 with mutations at R177 and S178. BW31 cells expressing variants of GFP‐*Hs*NHA2 from pGFP‐*Hs*NHA2*t* were grown in YNB‐Pro (4% glucose) to the exponential phase and observed under a fluorescence microscope (A, right). A Nomarski prism was used for whole‐cell imaging (A, left). In (B), protein extracts of cells were prepared as described in the Materials and methods section, subjected to SDS/PAGE (10% gel) and transferred to a nitrocellulose membrane. Native GFP‐*Hs*NHA2 and its variants were detected with an anti‐GFP antibody. The membrane was probed again with an anti‐Pgk1 antibody to verify the amount of loaded proteins. Cells expressing the native GFP‐*Hs*NHA2 or the version with P246T mutation were used as controls.

All antiporter's versions were expressed in cells under the same conditions (plasmid, promoter, terminator). To verify whether either of the modifications affected the protein stability and plasma membrane targeting of *Hs*NHA2, we next examined their localization and the amounts of corresponding synthesized proteins. As shown in Fig. 4A, all four single‐mutated versions were predominantly localized to the plasma membrane, similarly to the native GFP‐*Hs*NHA2 (Fig. 4A). On the other hand, the double‐mutated R177A + S178A version tagged with GFP was not properly targeted to the plasma membrane, and the observed signal indicates that the protein is retained in the perinuclear ER (Fig. 4A). We also detected the lowest signal of GFP‐*Hs*NHA2(R177A + S178A) protein in cells by immunoblotting (Fig. 4B). The immunodetection of single‐mutated versions (Fig. 4B) showed that mutations of S178A/T had little effect on the amount of the protein in cells, as the signal of these mutated GFP‐*Hs*NHA2 proteins was the same as for the native GFP‐*Hs*NHA2 or its P246T version, which were used as controls (Fig. 4B). On the other hand, although the same amount of proteins was loaded (detected with anti‐Pgk1 antibody), compared to the native GFP‐*Hs*NHA2, similarly decreased signals were observed for both versions with substitutions at position R177 (Fig. 4B).

All these results confirmed R177 and S178 to be important residues for *Hs*NHA2 functionality. With S178, the presence of a polar‐branched side chain at this position preserved the activity of the antiporter, but with higher capacity to transport Li^+^ than Na^+^ cations. The replacement of a side chain of R177 resulted in antiporters with the same substrate specificity as the native *Hs*NHA2 for Na^+^ and Li^+^. However, this residue also seems to be important for the structural integrity of the protein. Modifying it resulted in lower steady‐state levels of the antiporter, and hence a lower ability of cells to eliminate toxic cations, which correlates with reduced growth of cells expressing these versions of *Hs*NHA2 in the presence of salts. The predominantly intracellular localization and decreased stability of GFP‐*Hs*NHA2 with the R177A + S178A double mutation explained the inability of this variant to increase cellular salt tolerance (Fig. 3).

### Mutations R177T and S178T have opposite effects on the phloretin inhibition of *Hs*NHA2

**Fig. 5:**
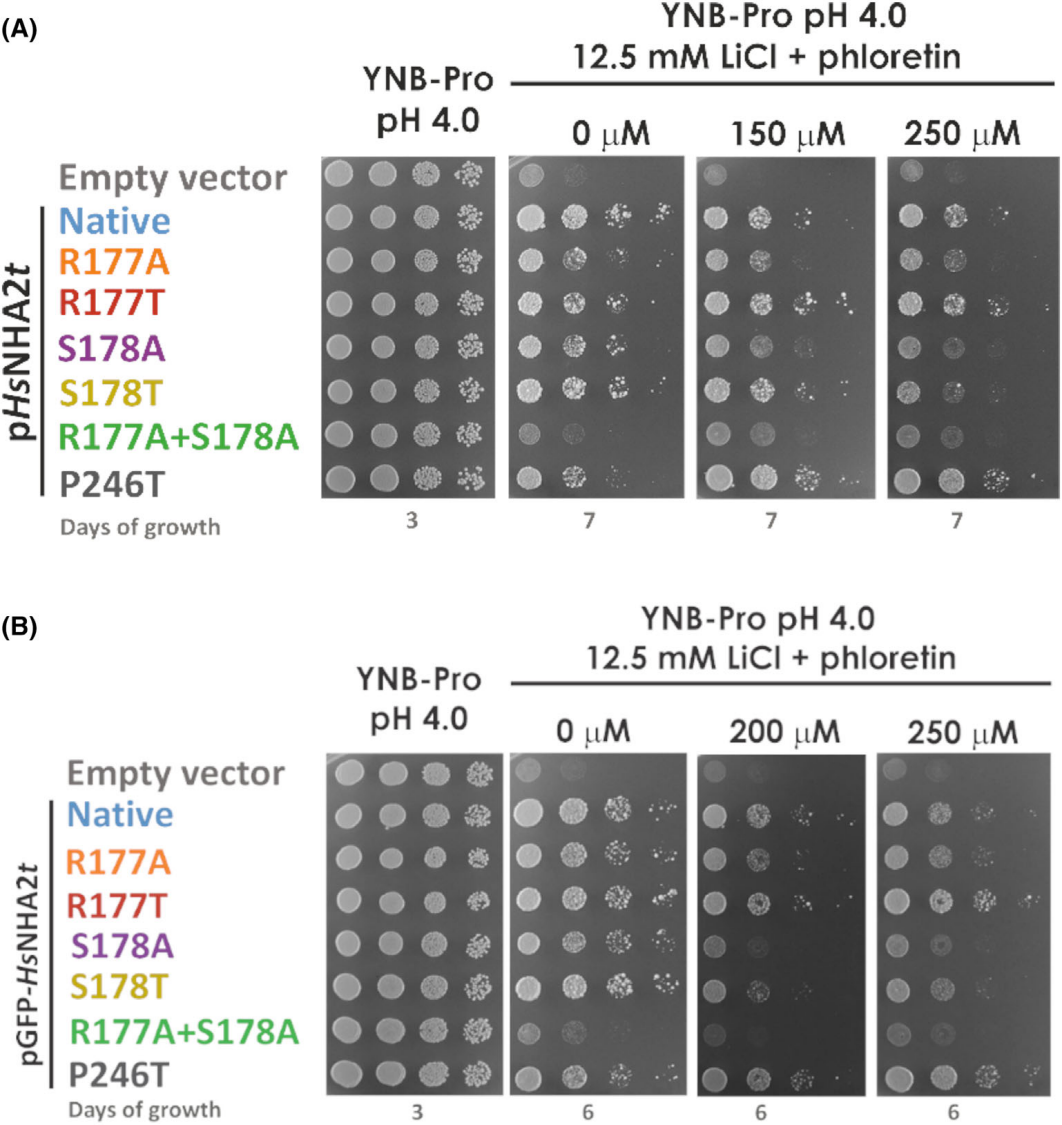
*Hs*NHA2 variants of R177 and S178 exhibit different sensitivities to phloretin inhibition. *S. cerevisiae* BW31 cells containing the empty vector or expressing native *Hs*NHA2 or *Hs*NHA2 versions with point mutations R117A, R177T, S178A, or S178T or the double mutation R177A + S178A from p*Hs*NHA2*t* (A) or pGFP‐*Hs*NHA2t (B) plasmids, as indicated, were used in this experiment. The inhibition of mutated *Hs*NHA2 versions by phloretin was determined by the growth of cells on YNB‐Pro plates with the pH adjusted to 4.0 and supplemented with LiCl, and with or without phloretin at the indicated concentrations. Plates were incubated at 30 °C and photographed on the indicated day. The experiment was repeated five times, and representative results are shown. Cells expressing the phloretin‐resistant variant of *Hs*NHA2 or GFP‐*Hs*NHA2 with the P246T mutation were used as controls.

The inhibition of *Hs*NHA2 by phloretin upon its expression in yeast cells was previously clearly demonstrated by drop tests of cells expressing *Hs*NHA2 in the presence of salts and increasing concentrations of phloretin [6, 22]. We used a similar approach in this work to determine the effect of mutations R177A/T and S178A/T on the sensitivity of the antiporter to phloretin. The ability of R177A and S178A versions of *Hs*NHA2 to improve the tolerance to NaCl was lower than on plates with LiCl (when comparing the growth with cells with the empty vector) (Fig. 3). So, we next tested the growth of cells harboring either the empty vector, native *Hs*NHA2 or mutated versions on plates supplemented with LiCl and increasing concentrations of phloretin (Fig. 5). In accordance with previous results [22], the ability of the native *Hs*NHA2 to improve the LiCl tolerance of yeast cells at pH 4.0 decreased in the presence of phloretin (Fig. 5A). On the other hand, the previously characterized phloretin‐less‐sensitive variant of *Hs*NHA2 with the P246T mutation improved the growth of cells to LiCl similarly in the absence or presence of phloretin (Fig. 5; [22]). In the same way, the antiporters with the replacement of R177, particularly R177T, were able to improve the cell tolerance on plates with LiCl and phloretin to the levels similar to those on plates with LiCl, but without phloretin (Fig. 5A). In contrast, with the replacement of S178 with alanine or threonine (S178A/T), the antiporter became more sensitive to phloretin than the native *Hs*NHA2 (Fig. 5A). Cells expressing *Hs*NHA2 with S178T grew only slightly better than cells with the empty vector on plates with 12.5 mm LiCl and 250 μm phloretin (Fig. 5A). Tagging of the N terminus with GFP did not change the level of sensitivity of particular *Hs*NHA2 versions to phloretin (Fig. 5B).

Together, our results confirmed the importance of both R177 and S178 for the proper activity of *Hs*NHA2, and show that both residues in TMS4 participate in a mechanism of inhibition mediated by phloretin.

## Discussion

Mammalian Na^+^/H^+^ antiporters of the SLC9 family mediate the exchange of Na^+^ and H^+^ across cell membranes, and thus play pivotal roles in the regulation of cell volume, organellar and cytosolic pH, and contribute to the monovalent cation homeostasis of the whole organism [3]. Each isoform of human Na^+^/H^+^ antiporters has been associated with various types of serious diseases [3], therefore understanding the molecular mechanisms governing ion transport and the binding of inhibitors is important in the development of therapeutics targeting particular NHE/NHA antiporters. Although these transporters can undergo a number of e.g., post‐translational modifications specific to human cells that can influence their functions [3], the cheap and relatively simplified experimental model based on their expression in a unicellular eukaryotic *S. cerevisiae* has been used for a long time to obtain valuable data about the structure, function, and mechanisms of inhibition of several isoforms of human Na^+^/H^+^ antiporters, including *Hs*NHA2 (e.g., [6, 10, 19, 20, 30, 31, 32, 33]). This work provides new data on the functioning of *Hs*NHA2 and extend current knowledge on the mechanism of inhibition of this antiporter by phloretin.

Our experiments demonstrate that amino acids R177 and S178, predicted by molecular modeling *in silico* (Fig. 2B), are indeed involved in the binding of the phloretin molecule. Mutations of both amino acid residues R177 and S178 also altered the activity of *Hs*NHA2 (Fig. 3). Nevertheless, with the R177A/T mutations, the reduced activity was probably due to lower stability, i.e., steady‐state levels, of the protein (Fig. 4). In contrast, the replacement of S178 with threonine did not affect the localization or stability of the antiporter in cells, and resulted in the same or slightly increased *Hs*NHA2 activity (Fig. 3). The presence of hydrophobic alanine instead of S178 negatively influenced the *Hs*NHA2 activity, particularly for Na^+^ cations, but also with no effect on stability or localization (Figs 3 and 4). This suggests that the polar character of the side chain at the S178 site is important for the transport of cations through *Hs*NHA2 and that this residue influences the substrate specificity of the antiporter. Consistent with the molecular modeling (Figs 1B and 2), all our experimental results also point to the involvement of side chains of individual amino acid residues in the cation pathway in the determination of substrate specificity and translocation of particular cations in human Na^+^/H^+^ antiporter NHA2, similarly as we described for the yeast plasma membrane Na^+^/H^+^ antiporters Nha/Sod [34, 35].

In the 3D structure of *Hs*NHA2, both R177 and S178 in TMS4 are positioned in the binding pocket on the opposite side to the cation‐binding site D279 (TMS7) and P246 (TMS6) (Fig. 2B), the latter when mutated was previously found to reduce phloretin sensitivity of *Hs*NHA2 ([22]; Fig. 5). The R177T or S178T mutations impacted the sensitivity of *Hs*NHA2 to phloretin in the opposite direction (Fig. 5). We generated models of phloretin binding to *Hs*NHA2 with the R177T and S178T point mutations (Fig. 2C,D, respectively). Based on this bioinformatic modeling, we hypothesize that the introduction of threonine at the R177 site disrupts cation‐π bonding interactions between the 2,4,6‐trihydroxyphenyl ring in the phloretin molecule and the arginine side chain (Fig. 2C). This is consistent with the results from drop test assays showing the lower sensitivity of the mutant version of R177T to phloretin (Fig. 5). The reason for the higher sensitivity of *Hs*NHA2 to phloretin upon introduction of the S178T mutation may be based on that the threonine side chain could adopt a different rotamer that could form hydrogen bonds with phloretin better than native *Hs*NHA2 (Fig. 2D). Therefore, the binding of a phloretin molecule to *Hs*NHA2 with the S178T mutation should not be weaker than binding to the native *Hs*NHA2 (Fig. 2D). Accurately theorizing why this mutation results in increased binding requires precise energy calculations by molecular dynamics methods that are beyond the scope of this work.

In summary, this study confirms that phloretin acts directly in the core domain of human NHA2 and that modifications at positions R177 and S178 change the transport properties and also the sensitivity of *Hs*NHA2 to this specific inhibitor, most likely by altering the binding site's geometry near the substrate/inhibitor‐binding pocket. Our computational study, supported by experimental evidence, provides a detailed mechanistic interpretation of phloretin‐*Hs*NHA2 interactions and may serve as the basis of future structure‐based inhibitor design to develop new classes of therapeutics targeting the *Hs*NHA2 antiporter and related pathologies.

## Author contributions

Conceived and Designed Experiments: OZ; Performed the experiments: OZ, MK, TP, GM. Analyzed the Data: OZ, GM. Drafted the Article: OZ; Writing—review and editing: OZ, GM; Prepared the Digital Images: OZ, MK, TP, GM.

### Peer review

The peer review history for this article is available at https://www.webofscience.com/api/gateway/wos/peer‐review/10.1002/1873‐3468.15089.

## Data Availability

The data that support the findings of this study are available from the corresponding author upon reasonable request.
